# Evaluation of hand hygiene implementation in hospitals in the post-war Tigray region of Ethiopia, using the WHO Hand Hygiene Self-Assessment Framework

**DOI:** 10.1016/j.infpip.2025.100490

**Published:** 2025-10-31

**Authors:** Akeza Awealom Asgedom, Bente Elisabeth Moen, Ana Lorena Ruano

**Affiliations:** a Department of Environmental Health Sciences, School of Public Health, College of Health Sciences, Mekelle University, Mekelle, Ethiopia; b Centre for International Health, Department of Global Public Health and Primary Care, University of Bergen, Bergen, Norway

**Keywords:** Hand hygiene, Health facilities, Tigray, Ethiopia, Post-conflict setting

## Abstract

**Background:**

Healthcare-associated infections are a major cause of morbidity and mortality among health personnel worldwide. The World Health Organization's (WHO) multi-modal hand hygiene tool has shown inadequate hand hygiene levels in various sub-Saharan countries. We have applied it here to describe the hand hygiene level in public health facilities in Tigray, Ethiopia.

**Methods:**

A cross-sectional study was conducted from June to July 2024 in all accessible public health facilities. An interview-based WHO hand hygiene self-assessment framework (HHSAF) tool was used for data collection. Descriptive analyses and independent *t*-test were used to analyse data.

**Results:**

A total of 33 facilities (two referral, 10 general, and 21 primary hospitals) from six accessible zones of Tigray participated in the survey. The mean age of the respondents was 35 years (SD: 8) with a mean service duration of nine years (SD: 7). Most respondents were infection prevention and control (IPC) focal persons (66.7%), followed by chief executive officers (9.1%). The overall HHSAF score was 126 (range: 15–318), indicating a basic hand hygiene level. Seventeen facilities (51.5%) had inadequate hand hygiene levels, 13 (39.4%) had basic hand hygiene level, three (9.1%) had an intermediate hand hygiene level, and none had an advanced hand hygiene level.

**Conclusions:**

Hand hygiene levels were unsatisfactory in post-war Tigray and were limited across all zones and types of facility, posing an increased risk of infection for healthcare personnel. Improvements in hand hygiene practices and IPC capacity building are essential to prevent healthcare-associated infections. Longitudinal research on hand hygiene level monitoring is recommended.

## Introduction

Healthcare-associated infections (HAIs) are a serious global threat to healthcare safety, causing sickness, death, and adding billions of dollars to healthcare costs each year, while also contributing to the rising levels of antimicrobial resistance (AMR) [1,2]. The associated human and financial costs of HAIs for health systems and the population are especially difficult to estimate when they take place during armed conflict [3]. An armed conflict occurred in the Tigray region between November 2020 and November 2022 resulting in extensive damage to civilian infrastructure. Numerous schools, private businesses, and public health services including hospitals were subjected to shelling, looting, and destruction [[4], [5], [6]]. There is a brief study indicating that healthcare workers lacked medical supplies during the conflict [7]. This led to patient and healthcare workers resorting to inappropriate IPC measures such as reusing gloves; using white cloth brought in by patients as gauze [8]; delivering new-borns with bare hands; and dressing wounds with unprotected hands; putting both themselves and patients at risk of infection [7].

HAIs pose a significant burden in Sub-Saharan Africa and other developing countries [9]. Evidence from a limited number of studies in the region indicates a widespread prevalence of HAIs with a pooled point prevalence of more than twice of those reported in higher-income settings [10,11]. Furthermore, there is notable heterogeneity in prevalence rates across different regions [1].

Implementing infection prevention and control (IPC) provides an effective solution for mitigating the risk of HAIs and AMR, as it reduces the risk of infection during delivery of essential health services [[12], [13], [14], [15]]. IPC interventions are recognized as both effective and highly cost-effective strategies. They represent a ‘best buy’ approach to reducing infections and combating AMR in healthcare settings, offering a significant return on investment [15]. Leadership and resource allocation play a crucial role in effectively implementing IPC guidelines and in reducing HAIs [16,17]. Extreme resource-constrained settings such as those experiencing conflict in Sub-Saharan Africa require significant IPC support programmes, as they help to control and deal with the secondary infections and help to improve the standard of care overall [14]. The World Health Organization's (WHO) multi-modal hand hygiene improvement strategy has been shown to be simple and cost effective, significantly improving key hand hygiene performance indicators, reducing HAIs and AMR [18]. The implementation of the WHO's multi-modal hand hygiene strategy in Sub-Saharan Africa has been summarily studied, and results show unsatisfactory hand hygiene levels [[19], [20], [21]]. There are no studies focusing on the hand hygiene levels of public healthcare facilities in the context of the conflict and subsequent fallout in Tigray. This study presents evidence useful for post-conflict interventions around IPC, including those that deal with HAIs and AMR. Its strength lies in the data from 33 public health facilities, representing the universe of facilities in the six accessible zones in Tigray, Ethiopia. One zone was not accessible due to security reasons.

## Methods

### Study setting description

Tigray is located in northern Ethiopia, has a population of six million people, and is divided into seven zones and 93 woredas, of which six zones are currently accessible [22]. A list of all health facilities for the state was used to identify hospitals, which were then contacted by the researcher. A support letter written by the Tigray Regional Health Bureau was presented to the facility management, and permission to conduct the study was requested and obtained. This study includes data from two tertiary referral hospitals, 14 general hospitals, and 24 primary hospitals. These facilities provide promotive, preventive, curative, and rehabilitative services. The study was conducted in the accessible Tigray zones, namely Southern, Southeast, Mekelle, Eastern, Central and Northwest, while the Western zone was inaccessible and was not included in the study. In the western zone, there are two primary hospitals and two general hospitals for which data was not collected.

After permission had been obtained, data was collected between June and July 2024 by environmental health professionals who are directly engaged as expert or focal persons in IPC-WASH activities in the healthcare setting under the direct supervision of the researcher. The head of each health facility (medical director or chief executive officer), IPC focal person, and matron (head of nurses) were respondents of the study. One person was interviewed per hospital.

### Study design

A cross-sectional descriptive study design was applied to describe the hand hygiene level of the facilities.

### Questionnaire description

The participants of the study were interviewed using the standard WHO-developed Hand Hygiene Self-Assessment Framework (HHSAF) tool. The HHSAF questionnaire is divided into five sections with 27 indicators [23]. The components are system change (six questions); training and education (five questions); evaluation and feedback (five questions); reminder in the workplace (five questions); and institutional safety climate for hand hygiene (six questions). Each section has a maximum score of 100 points, for a total maximum of 500, where higher scores are best. The hand hygiene promotion and practice level of the facility is classified into four categories based on the average score as shown in Table I.

**Table I:** Total Hand Hygiene Self-Assessment Framework score categories and their interpretations [23]

Total score (range)	Hand hygiene level	Interpretation
0–125	Inadequate	Indicates insufficient hand hygiene practices and promotion and requires significant improvement
126–250	Basic	Indicates that some measures are in place but not satisfactory and therefore requires further improvement
251–375	Intermediate (or consolidation)	Indicates appropriate hand hygiene promotion strategies and improvements in hand hygiene practices but requires long-term planning to ensure continual improvement and progress
376–500	Advanced (or embedding)	Indicates sustained hand hygiene promotion and practice as well as a quality and safety culture surrounding hand hygiene promotion within the organization

### Data analysis

Descriptive statistics were performed. Independent t-tests were used to compare the mean HHSAF values of the different types of health facilities. Significance level was set to *P* < 0.05. The Statistical Package for the Social Sciences (SPSS) version 25 was used for the statistical analyses.

## Results

### Demographic information

Out of a total of 35 eligible public health facilities, 33 facilities (two tertiary referral hospital, 10 general hospital and 21 primary hospitals) from the six accessible zones in Tigray participated in this study, with a response rate of 94%. Two hospitals were excluded from the study due to the insecurity of the conflict, which represented challenges to the safety of our data collectors.

The mean age of the respondents was 34 years (SD: 9) with an average seniority of nine years (SD: 7). Most of the respondents were men (69.7%). Many of the respondents were IPC focal persons (66.7%), and 9.1% were chief executive officers. The remaining respondents were medical directors, matrons or head of nursing, or a delegate nurse, representing 24.2% of the respondents.

### Total HHSAF score

**Figure 1:**
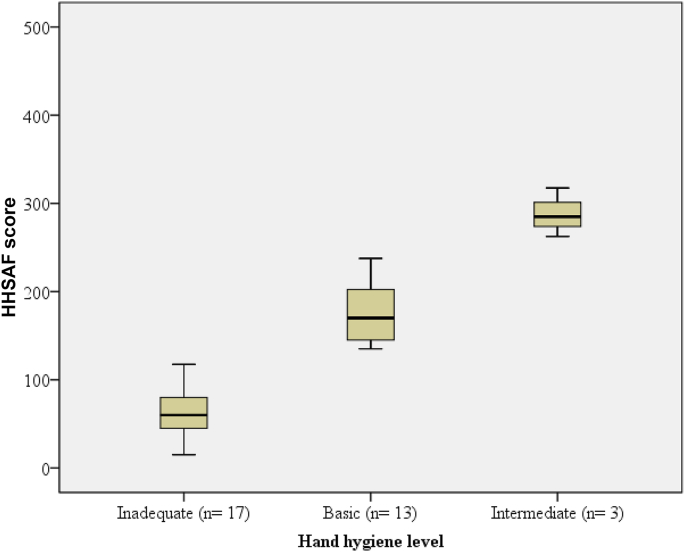
Hand hygiene level in 33 public hospitals across Tigray, Ethiopia.

The overall mean HHSAF score summarized for all facilities was 127 (range: 15–318), which is categorized as a basic hand hygiene level (see Table II). Of the 33 facilities, 17 (51.5%) had inadequate hand hygiene levels, 13 (39.4%) had basic hand hygiene levels, three (9.1%) had an intermediate (or consolidation) hand hygiene level, and no facilities had advanced (or embedding) hand hygiene level (Figure 1).

**Table II:** Hand Hygiene Self-Assessment Framework (HHSAF) scores of 33 public health facilities in Tigray, Ethiopia

Component	HHSAF score, mean (SD)				*P*-value^a^		
	Tertiary referral hospital (RH) (*N* = 2)	General hospital (GH) (*N* = 10)	Primary hospital (PH) (*N* = 21)	Total (*N* = 33)	RH vs GH	RH vs PH	GH vs PH
System change	10 (0)	12 (7)	16 (15)	14 (13)	0.695	0.590	0.437
Training and education	28 (18)	36 (24)	25 (20)	29 (21)	0.645	0.878	0.195
Evaluation and feedback	46 (41)	35 (20)	19 (20)	26 (22)	0.547	0.093	0.044^a^
Reminder in the workplace	61 (16)	38 (23)	25 (22)	31 (24)	0.201	0.040^a^	0.177
Institutional safety climate for hand hygiene	20 (28)	33 (26)	22 (25)	25 (25)	0.546	0.899	0.301
Total (N=33)	165 (103)	155 (83)	110 (77)	127 (81)	0.876	0.355	0.152

The maximum and best score for each component is 100, and 500 for the total score [23].

SD= Standard deviation.

a Comparison of groups using independent *t*-test.

### System change

This core component evaluates availability of alcohol-based hand rub in the healthcare facility, sink to bed ratio, availability of continuous supply of clean and running water, availability of soap and single-use towels at each sink, dedicated/available budget for the continuous procurement of hand hygiene products.

The mean HHSAF score for system change was 14 (SD: 13), representing the lowest value recorded. Fourteen facilities lacked access to alcohol-based hand rubs, while 17 had a sink-to-bed ratio of less than 1:10. Additionally, 24 facilities did not have a continuous supply of clean, running water.

Soap was absent in 29 facility sinks, and 30 facilities lacked single-use towels at each sink. Moreover, there was no dedicated budget allocated for the continuous procurement of hand hygiene products, such as alcohol-based hand rubs.

### Training and education

This core component evaluates frequency of healthcare workers' training on hand hygiene, availability of WHO documents on hand hygiene, availability of skilled professionals to serve as trainers in the facility, system for training and hand hygiene compliance, and availability of dedicated budget for hand hygiene training.

The mean HHSAF score for training and education was 29 (SD: 21). In 14 facilities, healthcare workers had never received training on hand hygiene. Additionally, none of the 33 facilities had a dedicated budget allocated for hand hygiene training.

### Evaluation and feedback

This core component evaluates regular annual ward-based audits for hand hygiene resources, healthcare worker knowledge assessment on hand hygiene, direct and indirect monitoring of hand hygiene compliance, and feedback on hand hygiene.

The mean HHSAF score for evaluation and feedback was 26 (SD: 22). Twenty-two facilities reported that no regular or annual ward-based audits were conducted to assess the availability of hand rub, soap, single-use towels, and other hand hygiene resources. Thirteen facilities displayed information on hand hygiene indications and proper techniques. Only eight reported indirect monitoring of hand hygiene compliance, such as tracking the consumption of alcohol-based hand rub and soap regularly at least every three months. Fourteen facilities had never conducted direct observation of hand hygiene compliance using the WHO hand hygiene observation tool or any other method, while another 14 reported irregular hand hygiene compliance observations. Only five facilities performed direct observations of hand hygiene compliance using the WHO Hand Hygiene Observation Tool. Additionally, 19 facilities reported a hand hygiene compliance rate of <30%.

### Reminder in the workplace

This core component evaluates hand hygiene strategies such as displaying hand hygiene posters, conducting systematic audits to check for damage and ensure timely replacement, and distributing promotional materials like information leaflets. Additionally, it includes other workplace reminders such as hand hygiene campaign screensavers, badges, and stickers.

The mean HHSAF score for reminders in the workplace was 31 (SD: 24). Nine facilities did not have posters displayed to explain the indications for hand hygiene, the correct use of hand rub, and proper handwashing techniques.

Only seven facilities conducted systematic audits of all posters to check for damage, with replacements made as needed. Hand hygiene promotion activities were present in just seven of the assessed facilities, and hand hygiene information leaflets were available in only ten facilities.

Additionally, only six facilities had workplace reminders distributed throughout the facility, such as hand hygiene campaign screensavers, badges, and stickers.

The reminders in the workplace received a higher score compared to other components, indicating that this approach is more effectively implemented. These reminders are relatively straightforward to apply.

### Institutional safety climate for hand hygiene

This core component evaluates the establishment of hand hygiene team, commitment for hand hygiene improvement, clear plan on ‘Save Lives Clean Your Hands’ annual initiative on May 5^th^, system for identification of hand hygiene leaders from all disciplines, patient involvement on hand hygiene promotion, and initiatives to support local continuous improvement being applied in the facility.

The mean HHSAF score for the institutional safety climate for hand hygiene was 25 (SD: 25). While 14 facilities had established hand hygiene teams, regular meetings conducted at least monthly were held in only four of them.

Additionally, only seven facilities had a clear plan for promoting hand hygiene across the entire facility as part of the May 5^th^ ‘Save Lives: Clean Your Hands’ annual initiative [24].

### Comparing facilities

When the mean HHSAF score was calculated by category of facility, inadequate hand hygiene level was found for the primary hospitals. Tertiary referral and general hospitals were generally found to have basic hand hygiene levels. Comparing the three groups of hospitals, there was no statistically significant difference between them regarding the total HHSAF (*P* > 0.05) (Table II). However, there was a significant difference among tertiary referral and primary hospital regarding specific components of HHSAF. The component ‘reminder in the workplace’ had a lower score in primary hospitals compared to tertiary referral hospitals. In addition, the score for the hand hygiene component ‘evaluation and feedback’ was significantly lower in primary hospitals compared to general hospitals (Table II).

### Comparing zones

**Figure 2:**
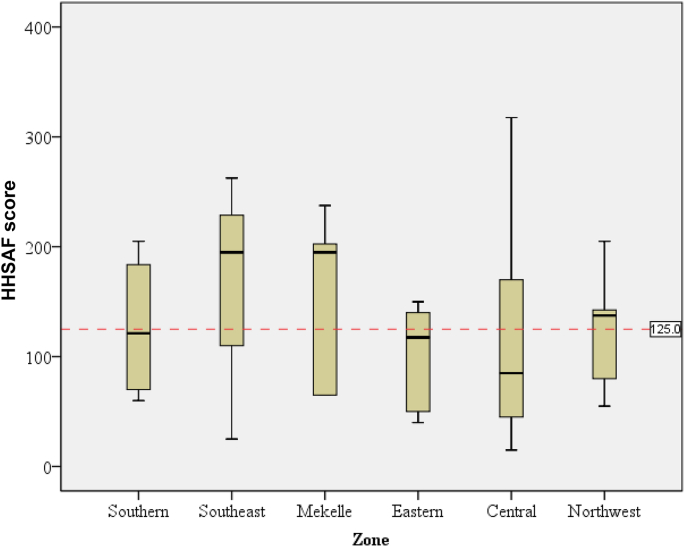
Total Hand Hygiene Self-Assessment scores in the public health facilities in six different geographical zones of Tigray, Ethiopia. The box plots show the medians, quartiles, minimum, and maximum hand hygiene scores in the facilities. The red dashed line indicates a cut point of 125, indicating inadequate hand hygiene level below the line. Maximum and best score is 500 [23].

When the HHSAF score was analysed by the six zones studied, an inadequate HHSAF score was found in three of them: Eastern: 98 (SD: 50); Central: 124 (110); and Northwest: 124 (59). The HHSAF value ranged from 15 to 318 (Figure 2). Basic level was found in the Southern zone: 127 (68); Southeast: 161 (122); and Mekelle: 153 (82) zones, indicating that some measures are in place although they are not satisfactory.

## Discussion

The total mean HHSAF score from 33 public health facilities of the war-affected state of Tigray was 127. This is a very low score, considering the scale's top score is 500. The scores for all five sub-components were similarly low, indicating insufficient hand hygiene in all facilities included in our study.

The range of hand hygiene score in the study went from 15 to 318, and although they are wide-ranging, they can be categorized as basic hand hygiene levels and interpreted as unsatisfactory and even lower for the region. A similar study conducted in Sierra Leone found scores of 273 and 278 for 2021 and 2023, respectively [19]. Comparison with studies from higher-income settings like one conducted in Greece (score 289), Italy (score 332) and the USA (score 373) also show that facilities can routinely score higher if the health system's building blocks, including information systems, service delivery, leadership and governance, and financing, are in place and working connectedly [[25], [26], [27], [28]].

When it comes to global-level studies on hand hygiene, findings from a 69-country study showed a mean result of HHSAF of 335 in 2011, while a 91-country study from 2015 found a mean score of 374, and another 90-country study found a median of 350 in 2019 [29,30]. The data shows that the income level played an intermediary role for higher scores and allowed a higher classification of hand hygiene levels [30]. It is likely that the low score found in our study is related to the conflict experienced by the region, as the war has affected not only the health infrastructure, but also the economy and general infrastructure around the IPC facilities in Tigray. This resulted in low levels of hand hygiene. It is worth noting that existent scholarship does not reflect conflict or post-conflict contexts and how they affect IPC, AMR, and HHSAF more broadly. Future studies should focus on the impact of conflict on HHSAF scores and on qualitative studies to understand how the health workforce makes decisions regarding hygiene policies and what can be implemented under duress. However, findings from a study conducted in conflict-affected Syria showed that the majority of facilities had significant deficits in hand hygiene compliance, which is in line with our findings [31].

During the conflict in Tigray, many health facilities were attacked, and their basic infrastructure damaged. This resulted in non-existent IPC activities. According to the Health Resources and Services Availability Monitoring System (HeRAMS) assessment from Tigray in 2023, 51% of 754 facilities assessed had no water services, 46% had no sanitation services, 74% lacked waste segregation, and 65% had no hand hygiene services [32]. A recent study on post-conflict Tigray on primary care facilities also indicated the limited status of water, sanitation and hygiene (WASH) initiatives and work [33]. This might have contributed to the observed low hand-hygiene level and reflects the low funding for IPC [30]. The already weak health system building blocks in Tigray most likely contributed to the IPC activities in healthcare facilities during the conflict.

When the hand hygiene level was analysed by each component in different hospitals, there was a significant difference among tertiary referral and primary hospital regarding ‘reminder in the workplace’. This indicates that primary hospitals show less compliance regarding the hand hygiene reminders in the workplace. There was also a statistically significant difference among general and primary hospitals regarding ‘evaluation and feedback’. This shows that general hospitals provide some evaluation and feedback relative to primary hospitals.

Most of the mean HHSAF scores for the different hand hygiene components were <50. This means the hand hygiene is very low and is likely to be a cause of HAIs. Leadership plays a crucial role in the effective implementation of hand hygiene compliance. By fostering a culture of safety, leaders can significantly contribute to the reduction of HAIs [16,34].

Looking at the scores by zones might help to parse out how lack of, for example, WASH infrastructure might have affected the outcome of our study. Community-based studies showed low access for WASH across zones in Tigray [35,36]. This might explain how access for WASH in the respective health facilities contributes to low hand hygiene levels. This has implications for HAIs, as studies indicate that WASH and interventions to improve hand hygiene compliance are inversely related with prevalence of HAIs [21,37,38]. This means that hand hygiene interventions are not only timely but sorely needed to improve and ensure quality services, but also to minimize HAIs in Tigray. Studies suggest that availability of resources, leadership, and organizational support are key elements to further improve quality of care and provide access to safe care for all [30]. Other studies indicated that despite a shortage of water and other resources, implementing the WHO hand hygiene improvement strategy significantly increased hand hygiene compliance [39]. Adherence to hand hygiene practices is consistently associated with a reduction in HAIs [37,40]. Furthermore, another study conducted in Tigray tertiary hospital indicated that multi-modal prevention strategies such as IPC training, appointment of an IPC expert, in-house production of alcohol-based hand rub (ABHR), conducting surveillance of healthcare-associated bloodstream infections (HA-BSIs), and holding online IPC meetings can significantly decrease HA-BSIs and related mortality rates in neonatal intensive care units in low-resource settings [41].

Due to the low levels of hand hygiene practice in healthcare facilities, it is likely that they negatively affect the quality of care that patients and communities receive in post-conflict Tigray. This calls for a strong policy focus on implementation and capacity building of IPC in healthcare facilities of Ethiopia, while also overall strengthening of all health system building blocks.

This study has some limitations. The cross-sectional design provides only a snapshot of the hand hygiene levels and cannot capture the complexity or entirety of this status. In addition, we did not have information about hand hygiene levels before the conflict, which limits our ability to interpret our findings more clearly. Despite this, our study is a census, not a sample, of all accessible public health facilities in Tigray, making the results representative for the entire region studied. Another strength is the use of WHO's standardized and tested HHSAF tool for data collection, which increases the validity of the data because it has been shown that this tool actually measures the hand hygiene and is validated in its usability and reliability [42]. However, the data was self-reported, and no objective measurements or observations were made. This could have caused information bias. We do not know whether the reports differed from the actual situation, but we have no reason to believe that there was specific information bias present in this study.

These results show that there is a need for improving hand hygiene in post-conflict settings such as Tigray. Resources are needed to build up infrastructure in the region's health facilities and should be prioritized to avoid even higher levels of hospital-acquired infections. This finding might help to expand the existing knowledge from conflict-affected low-income countries where available evidence is scarce. The data and our results might also be useful for planning of resources, priority setting exercises for improving equitable allocation of resources, and help in similar post-conflict situations elsewhere. Future longitudinal studies are essential to assess the level of hand hygiene.

## Conclusion

Hand hygiene levels were found to be unsatisfactory in all health facilities in all geographically accessible zones in post-conflict Tigray, posing an increased risk of HAIs. Improvements in hand hygiene, coupled with IPC capacity building, are essential to prevent HAIs. Furthermore, continuous monitoring of the hand hygiene levels must be implemented to track the trend of hand hygiene compliance, and this should be implemented using health systems and policy strategy to act on the structural and contextual factors contributing to the low levels of hand hygiene.

## Credit authorship contribution statement

**Akeza Awealom Asgedom:** Conceptualization, Data curation, Formal analysis, Methodology, Software. **Bente Elisabeth Moen:** Conceptualization, Funding acquisition, Methodology, Resources, Supervision. **Ana Lorena Ruano:** Formal analysis, Methodology, Supervision.

## Funding sources

This study was funded by the Norwegian Program for Capacity Building in Higher Education and Research for Development (NORHED-II Safeworkers Project), grant number: 69181.

## Ethical approval and consent to participate

Ethical clearance was obtained from College of Health Sciences, Institutional Review Board of Mekelle University (Ref. No: MU-IRB 2068/2023). A support letter from Tigray Health Bureau was obtained and given to the management of the facilities. Written informed consent from the health facility management was assured before the actual data collection. All research methods were performed in accordance with the ethical principles of the Declaration of Helsinki that assures beneficence, non-maleficence, autonomy, and justice.

## Conflict of interest statement

None declared.
